# Mixing two different propolis samples potentiates their antimicrobial activity and wound healing property: A novel approach in wound healing and infection

**DOI:** 10.14202/vetworld.2018.1188-1195

**Published:** 2018-08-29

**Authors:** Noori Al-Waili

**Affiliations:** 1 Private Clinic, Basic Science Research, Al-Rusafa, Baghdad, Iraq; 2 New York Medical Care for Nephrology, New York, 11418, US

**Keywords:** healing, microorganisms, mixed propolis, wound

## Abstract

**Aim:**

The study aimed to investigate whether mixing two different propolis samples can potentiate their biological activity. This hypothesis was tested by studying the effect of mixed propolis on microbial growth and wound healing and compared with the effect of each propolis individually.

**Materials and Methods:**

The effect of mixing two different propolis extracts (A and B) collected from different locations in Iraq on *Escherichia coli, Staphylococcus aureus*, and *Candida albicans* was studied by minimum inhibitory concentration assessment and compared with the effect of each propolis. Wound healing effect of the mixed propolis was studied. Twenty-four rabbits were used for the experiment, and they were assigned to four groups. Wounds were created on the dorsum of each rabbit and treated by topical application of 1 mL of either mixed propolis, propolis A, or propolis B extracts or were kept without treatment as a control. Macroscopic wound evaluation was performed with an assessment of wound size, wound recovery, redness, edema, discharge, granulation tissue, and epithelialization.

**Results:**

Propolis A was more potent than propolis B extracts to inhibit the growth of *E. coli, S. aureus*, and *C. albicans* (p<0.05). However, mixed propolis showed a higher antimicrobial activity toward all the pathogens than propolis A or propolis B extract individually (p<0.05). Furthermore, propolis A and propolis B extracts showed favorable effects on wound healing which was more pronounced with propolis A extract. Interestingly, mixed propolis accelerated wound healing faster than propolis A or propolis B extracts, and it shortened the time of reepithelialization (p<0.05).

**Conclusion:**

This study demonstrates for the first time that mixing different propolis samples possesses a higher antimicrobial activity and higher wound healing property than individual propolis. This approach could pave the way for the development of more effective antimicrobials and wound healing agents.

## Introduction

Honeybee produces propolis from buds and exudates that are modified by wax and bees’ salivary secretions. Propolis has been used in folk medicine for a long time. It has various potential biological activities that include anti-inflammatory, immunomodulatory, antitumor, antioxidant, radioprotective, antiproliferation, antidiabetic, antiproteinuric, and antimicrobial effects [1-9]. We found that propolis collected from the Arabian Peninsula or Egypt has potent antimicrobial activity against antibiotic-resistant *Staphylococcus aureus* and *Escherichia coli* and *Candida albicans*, and their effects were potentiated by honey [9].

Propolis enhanced wound healing in different animal models including diabetic wounds and burns [10,11]. We have found that the topical application of propolis significantly enhanced the closure of diabetic wounds, normalized the levels of interleukin (IL)-1β, IL-6, tumor necrosis factor alpha (TNF-α), and matrix metallopeptidase 9 (MMP9), and significantly enhanced the production of collagen through the transforming growth factor beta 1 (TGF-β1)/Smad2,3 signaling axis in wounded tissues [2]. In a clinical trial, 24 patients with diabetic foot ulcer were treated with topical propolis, and the results demonstrated the effective therapy of propolis for wound healing [10].

It has been reported that propolis has various chemical compounds that depend mainly on the geographical areas, the season, harvesting periods, and other environmental factors [12,13]. This is because honeybees gather propolis from various resinous parts of plants and in different phytogeographic regions. Its color varies from green to brown and reddish, depending on its botanical source. The main components of propolis are resin (including polyphenolic compounds) 50%, wax 30%, essential oils 10%, pollen 5%, and various organic and inorganic compounds 5%. Propolis contains volatile oils, terpenes, and bee wax. More than 300 chemical ingredients have been identified in propolis [14].

Propolis collected from tropical regions is rich in prenylated derivatives of *p*-coumaric acids, benzophenones, or terpenoids while propolis collected from temperate climatic zones is mainly rich in flavonoids and phenolic acids and their esters [14-16]. According to the chemical profiles and the plant sources, several propolis types are known such as Brazilian green propolis (*Baccharis* type), poplar-type (European) propolis, Brazilian red propolis (*Dalbergia* type), and Mediterranean propolis [17]. Brazilian propolis is characterized by the presence of derivatives of prenylated cinnamic acid [16,18].

Studies have shown that the activities of propolis, including its antibacterial activity, are dependent on the plant species, the harvesting periods, the geographical and climatic factors, and the type of bee species [19-22]. Propolis activities are more evident in tropical regions than in temperate climates [16]. Variations in propolis activities can occur among propolis samples collected in the same area but by different *Apis mellifera* subspecies and by different seasons [19]. Furthermore, recently, we have found that the antimicrobial property of propolis varies with geographical origin [9,23]. Therefore, the propolis samples collected from different regions have different chemical compositions, and the bioactivities are dependent on the propolis compositions.

We had hypothesized that, since propolis produced by honeybees from different plant sources, mixing different propolis samples gathered from different geographical areas or seasons or produced by different bee species might yield super propolis with better bioactivities. To test this hypothesis, the study investigated the antimicrobial activity and wound healing property of mixing two different propolis samples collected from different areas in Iraq; the data were compared with the effect of each propolis sample on wound healing and bacterial and fungal growth.

## Materials and Methods

### Ethical approval

All the experiments were conducted in accordance with the internationally accepted principles for laboratory animal use and care. All animal experiments were carried out under protocols approved by the Ethics Committee, Medical Clinic, Baghdad, Iraq (process 1/003).

### Propolis samples and extracts

Two types of propolis collected from different geographical areas in Iraq (multiplant forests) were studied; propolis A was collected during spring from Hillah, Babil Province, south of Baghdad, where date tree and crops are common, and propolis B was collected during summer form Dhiala where orange and grapes trees are common. Alcohol extraction of both propolis samples was performed. The propolis was crushed to make a powder, and then, 100 g of propolis A or propolis B was added to 1 L of 70% ethyl alcohol and kept in a beaker covered with aluminum foil for 7 days at room temperature with frequent shaking. The alcohol was evaporated, and the extract of propolis A and propolis B was weighed. Mixed propolis was prepared by mixing equal amount of propolis A and propolis B extracts. The extract was weighted and dissolved in nutrient broth to make a concentration of 5% (weight/volume), and various concentrations were made after dilution with nutrient agar (0.1-2.0%). Ethyl alcohol was evaporated before dilution in the nutrient broth to obtain a pure propolis/nutrient broth mixture.

### Effect of mixed propolis on microorganisms

Fresh cultures of human pathogens, isolated from chronic wounds, were used. The pathogens including *S. aureus*, *E. coli*, and *C. albicans* were obtained from the Microbiology Private Laboratory, Baghdad, Iraq, and the isolates were identified by the standard bacteriological techniques. Using a standard loop, a colony of each isolate was picked from the plate and was transferred into 10 ml nutrient broth. The broth culture was used after 24 h incubation at 37°C.

To study the antimicrobial activity of the propolis A, propolis B, and mixed propolis on the pathogenic isolates, specimen of each pathogen was taken from the pure culture grown in 10 ml nutrient broth and then was cultured in broth containing different concentrations of propolis A extract, propolis B extract, or mixed propolis to measure minimum inhibitory concentration (MIC). The different concentrations of propolis in the liquid broth (wt/volume) included 0.05%, 0.1%, 0.15%, 0.20%, 0.25%, 0.30%, 0.35%, 0.40%, 0.45%, and 0.50%.

After incubation at 37°C for 24 h, a loopful of the culture of each of the specimen microorganisms (control) and the cultures of each of the specimen microorganisms in broth containing various concentrations of each propolis sample were streaked onto agar plates, which were incubated aerobically at 37°C, and inspected after 24 h for microbial growth. Solid media including mannitol salt agar for *S. aureus*, MacConkey agar media for *E. coli*, and Sabouraud media for *C. albicans* were used.

Bacterial growth was assessed visually on nutrient agar plates (Oxoid, U.K.) as follows: 0 colonies = no growth, 1-5 colonies = little growth, 6-20 colonies = mild growth, 21-50 colonies = moderate growth, and >50 and uncounted colonies = heavy growth. The cultural media and materials were ready made and supplied by the Private Microbiology Laboratory, Baghdad, Iraq.

### Effect of mixed propolis on wound healing

Twenty-four adult male, white New Zealand rabbits weighing 1.8-2.2 kg were used for the experiment. Each animal was restrained in the clean and well-ventilated box. The animals were given access to water and were fed *ad libitum*.

The paravertebral region of each rabbit was shaved and cleaned. After anesthesia with intravenous thiopentone sodium (40mg/kg b.wt) using rabbit’s ear vein, the skin was cleaned with antiseptic 2% chlorhexidine, and a circular full-thickness wound was made with a 3-cm diameter on the dorsum of each rabbit aseptically. The wounds were made with the use of blades, forceps, and scissors. The wounds were left open to heal by secondary intention. Each propolis extract (1 mL) was administered topically to cover the entire wound area with the use of a sterile spatula.

**Figure-1:**
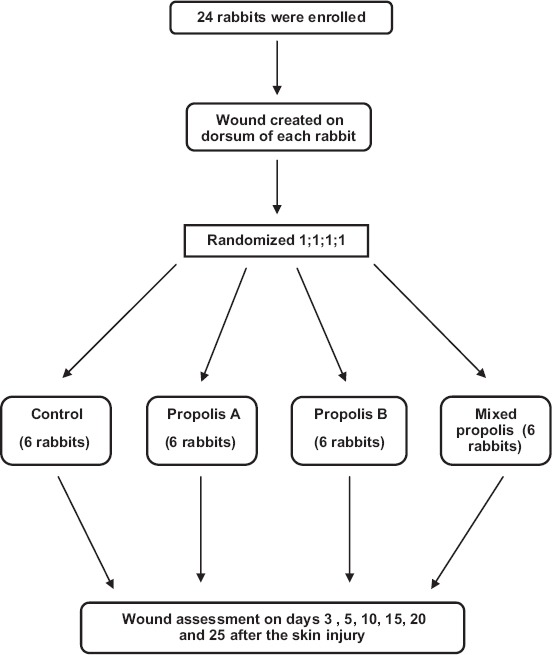
Clinical trial flow diagram.

The animals were divided randomly into four groups each, containing six rabbits; Group 1: No treatment, Group 2 was treated by topical application of propolis A, Group 3 was treated by topical application of propolis B, and Group 4 was treated by topical application of mixed propolis (Figure-1). Each propolis sample extract (1 mL) was applied to the wounds directly 2 times a day, 12 h apart. The wounds were washed with normal saline before application of propolis extract to remove residues and crusts.

Macroscopic assessment of the wound was made by examination for redness, edema, discharge, granulation, and epithelization. These wound healing parameters were ranked as follows: 0 (none), + 1 (mild or small), + 2 (moderate), and + 3 (severe or large). Mild and severe were used for grading redness, discharge, and edema, while small and large were used for grading granulation and epithelialization. Wounds sizes were evaluated planimetrically by a transparent sheet, and the area was measured by a graph paper (mm^2^). The percentage reduction in the wound size (wound recovery) was calculated using the following equation; percentage reduction = wound size on the 1^st^ day – wound size on the day* × 100 divided by wound size on the 1^st^ day; *day of wound size measurement. The wounds were examined for the parameters evaluation at 3, 5, 10, 15, 20, and 25 days after the skin injury.

A complete wound healing was considered macroscopically when the wound whole surface area was covered with epithelium. Any wound that developed signs of infection would be recused with antibiotics, and wound culture would be performed.

### Statistical analysis

The results were expressed as a mean values±standard deviation. Statistical analysis of the data was performed with ANOVA followed by Tukey’s test. Significant differences were indicated by p<0.05.

## Results

All the rabbits used in the experiments survived the initiation of the wounds, and no mortality was encountered during the study period. The propolis A and propolis B extracts inhibited the growth of *S. aureus, E. coli*, and *C. albicans*. Their MIC toward all the pathogens tested was significantly different (Table-1). Propolis A extract was significantly more potent than propolis B against all the three microorganisms (p=0.0117). However, mixed propolis (50% propolis A and 50% propolis B. wt/wt) inhibited all the pathogens, and its MIC toward all the three isolates was significantly lower than propolis A (p=0.0002) or propolis B (p=0.0000) individually. This indicated that mixed propolis is more potent than either propolis A or propolis B.

**Table-1:** Antimicrobial effects of single propolis or mixed propolis in human pathogens.

Type of propolis	Microorganism	Control	Propolis concentration % wt/v						MIC

			0.10	0.15	0.20	0.25	0.30	0.35	
Propolis A	*E. coli*	4+	3+	3+	0	0	0	0	0.20
	*S. aureus*	4+	3+	3+	1+	0	0	0	0.25
	*C. albicans*	4+	4+	3+	3+	2+	0	0	0.30
	Mean±SD								0.25±0.05
Propolis B	*E. coli*	4+	3+	2+	1+	1+	0	0	0.30
	*S. aureus*	4+	3+	3+	2+	1+	0	0	0.30
	*C. albicans*	4+	4+	3+	3+	1+	1+	0	0.35
	Mean±SD								0.31±0.02*
Mixed propolisA+B	*E. coli*	4+	2+	0	0	0	0	0	0.15
	*S. aureus*	4+	1+	0	0	0	0	0	0.15
	*C. albicans*	4+	2+	0	0	0	0	0	0.15
	Mean±SD								0.15±0*^#^

MIC=Minimum inhibitory concentration. F value is 40.552 andp value is 0.00.

* p<0.05 as compared to propolis A.

# p<0.05 as compared to propolis B. *E. coli=Escherichia coli, S. aureus=Staphylococcus aureus, C. albicans=Candida albic*ans, SD=Standard deviation

There was a significant reduction in the wound surface area in the propolis A group and propolis B group on days 10 and 15 compared to those in the control group (p<0.05) (Table-2). However, in the mixed propolis group, the reduction in the wound surface area was significantly higher on days 3, 5, 10, and 15 than that observed in the control group and significantly higher on days 5, 10, and 15 than that observed in the propolis A and B groups (p<0.05). Interestingly, the wound surface area in the Group A was significantly smaller on days 10 and 15 than that obtained in the propolis B group (p<0.05).

**Table-2:** Effect of propolis A, propolis B, and mixed propolis on wound size.

Time (days)	Wound size (cm^2^)				F/p values

	Control	Propolis A	Propolis B	Mixed propolis	
3	6.8±0.2	6.33±0.9	6.3±0.7	5.5±0.9*	3.22/0.044
5	6.3±1.2	5.3±0.8	5.8±0.8	3.8±0.5^#^*^+^	9.23/0.0004
10	5.4±0.9	2.1±0.4*	3.0±0.1^#^*	1.2±0.3^#^*^+^	73.17/0.000
15	2.3±0.3	0.5±0.3*	1.4±0.4^#^*	0.00	61.32/0.000
20	0.78±0.14	0.00	0.00	0.00	186.24/0.0000
25	0.00	0.00	0.00	0.00	

* p<0.05 as compared to the control.

# p<0.05 as compared to the propolis A.

+ p<0.05 as compared to the propolis B. 0.00=Complete wound healing and the whole wound covered by epithelium

Table-3 showed the percentage of wound recovery. Mixed propolis] caused significantly higher (p<0.05) percentage of wound recovery than the control group from the day after the surgery, while propolis A and B groups caused a higher percentage of wound recovery started on day 10 after the surgery. Furthermore, mixed propolis caused a significantly higher wound recovery than propolis A and B groups at days 5, 10, and 15 (p<0.05). The wound recovery was significantly higher in the group treated by propolis A than that in the group treated by propolis B. This indicated that propolis A was more potent than propolis B.

**Table-3:** Percentage of wound recovery (mean±SD).

Time (days)	Wound recovery				F/p values

	Control	Propolis A	Propolis B	Mixed propolis	
3	2.3±3.6	9.4±10.5	9.4±10.1	18.2±12*	3.10/0.049
5	9.4±12.1	19.4±14	17.4±13.2	47±14*^#+^	10.29/0.0003
10	24.1±12	70±6.6*	56.6±2.5*^#^	83±4.0*^#+^	69.9/0.0000
15	67.1±5.4	91±4.6*	78.6±3.6*^#^	100±0.00*^#+^	76.36/0.0000
20	88±2.2	100±0.00*	100±0.00*	100±0.00*	178.51/0.000
25	100±0.00	100±0.00	100±0.00	100±0.00	

* p<0.05 as compared to the control.

# p<0.05 as compared to the propolis A.

+ p<0.05 as compared to the propolis B. SD=Standard deviation

The wound reepithelialization was larger in the propolis group A or the propolis group B on days 5, 10, 15, and 20 compared to that observed in the control group (p<0.05) (Table-4). However, the differences were significant on days 10 and 15 with the use of propolis A. With the use of the mixed propolis, the wound reepithelialization was significantly larger on days 3, 5, 10, and 15 than those observed in the control group and larger than those observed in the propolis A or propolis B groups; the difference was significant on day 10 as compared to the propolis B group (p<0.05). No significant differences were noticed between propolis A group and propolis B group though the wound reepithelialization was larger in the propolis group A at 10 and 15 days after surgery.

**Table-4:** Effect of propolis A, propolis B or mixed propolis on wound redness, discharge, edema, granulation, and epithelialization.

Variables	Time (days)	Intervention				F/p values

		Control	Propolis A	Propolis B	Mixed propolis	
Redness	3	0.66±0.5	0.5±0.54	0.66±0.85	0.16±0.4	0.936/0.442
	5	1.66±0.51	0.66±0.51*	0.66±0.51*	0.0*	14.80/0.000
	10	0.5±0.51	0.0	0.0	0.0	2.95/0.057
	15	0.0	0.0	0.0	0.0	
	20	0.0	0.0	0.0	0.0	
	25	0.0	0.0	0.0	0.0	
Discharge	3	0.66±0.51	0.66±0.81	0.5±0.54	0.5±0.51	0.14/0.935
	5	1.66±0.51	0.5±0.54*	0.66±0.5*	0.0*	14.5/0.000
	10	0.83±0.75	0.166±0.4	0.5±0.54	0.0*	3.17/0.046
	15	0.16±0.4	0.0	0.0	0.0	1.033/0.399
	20	0.0	0.0	0.0	0.0	
	25	0.0	0.0	0.0	0.0	
Edema	3	0.83±0.4	0.66±0.51	0.66±0.51	0.5±0.51	0.45/0.718
	5	1.5±0.54	0.83±0.4	0.83±0.75	0.0*^#+^	8.925/0.001
	10	0.83±0.4	0.166±0.4*	0.5±0.54	0.0*	5.323/0.007
	15	0.166±0.4	0.0	0.0	0.0	1.03+/0.399
	20	0.0	0.0	0.0	0.0	
	25	0.0	0.0	0.0	0.0	
Granulation	3	0.0	0.0	0.0	0.0	
	5	0.0	1.83±0.75*	1.66±1*	2.16±0.4*	12.95/0.000
	10	1.5±0.54	2.3±0.81	2.3±51	1.66±1.0	1.934/0.157
	15	2.16±0.75	1.5±0.51	1.83±0.75	0.0*^#+^	15.725/0.00
	20	0.33±0.51	0.0	0.0	0.0	2.512/0.88
	25	0.0	0.0	0.0	0.0	
Epithelialization	3	0.0	0.0	0.0	0.5±0.54*^#+^	5.144/0.008
	5	0.66±0.4	1.0±0.63	1.0±0.36	1.6±0.54*	3.752/0.027
	10	1.33±0.51	2.33±0.51*	1.83±0.4	2.6±0.51*^+^	8.44/0.0008
	15	2.16±0.5	2.8±0.4*	2.6±0.5	3.0±0.0*	4.69/0.012
	20	2.8±0.4	3.0±0.0	3.0±0.0	3.0±0.0	1.5/0.245
	25	3.0±0.0	3.0±0.0	3.0±0.0	3.0±0.0	

* p<0.05 as compared to the control,

+ p<0.05 as compared to propolis B.

# p<0.05 as compared to propolis A

The amount of granulation tissue was larger in the propolis A group and propolis B group on days 5 and 10 as compared to the control though the differences were not significant. However, in the mixed propolis, the granulation tissue was larger on day 3 than all other groups, but the amount of granulation was lesser on days 5, 10, 15, and 20 than the control group and on days 10 and 15 than that observed in the propolis A group or propolis B group (p<0.05).

Regarding the other parameters, propolis A, propolis B, or mixed propolis caused less redness, discharge, and edema compared to the control group and mixed propolis caused less redness, discharge, and edema compared to the propolis A or propolis B (Table-4). The discharge observed in all the groups was serosanguineous.

The time needed for the complete reepithelialization and wound healing in the mixed propolis group was 15 days which was shorter than that needed for the complete reepithelialization and wound healing in the propolis A group (20 days), propolis B group (20 days), and the control group (25 days).

In the control groups, the wounds showed signs of infections with pus formation in two rabbits on days 6 and 8 and were treated with antibiotics. No wound treated with propolis A, propolis B, or mixed propolis showed sign of infection, pus formation, or required treatment with antibiotics.

No side effects were recorded after application of propolis extracts on the wound surface during the whole period of the study. All the rabbits survived until the end of the study with no signs of infection.

The center of the treated wounds became a scar after 10-12 weeks of the wound initiation. The scar size in all propolis-treated groups was smaller than scar size in the control group, and it was the smallest in the mixed propolis group.

## Discussion

The two different propolis samples exhibited antimicrobial activity against Gram-positive and Gram-negative bacteria isolated from chronic wounds, as well as *C. albicans*, but with different potency. The two samples were collected from different areas and seasons. These two areas were different in predominate plants. Both samples have potent antimicrobial activity, but propolis A has higher activity than propolis B. Mixing the two samples provided new propolis with a higher antimicrobial potency compared to each propolis. This is an important finding because it is possible to get new propolis with higher biological activities by mixing two or more than two different individual propolis samples collected from various geographical regions or different seasons or mixing samples gathered by different bee species. This method will overcome future microbial resistant to certain types of propolis by mixing it with another type of propolis to yield highly effective propolis. This approach is supported by the fact that propolis samples are different in their actions and their chemical compositions. There was no previous study investigated the synergism or additive antimicrobial activity of mixing different propolis samples.

Various propolis samples have different potencies to inhibit microbial growth. It was found that German propolis inhibits *S. aureus* and *E. coli* and Austrian propolis inhibits *C. albicans* [24]. Another study revealed that propolis from the Netherlands and China possessed the strongest cytotoxic activity as compared to propolis from Brazil or Peru [25]. Furthermore, propolis collected from Arabian Peninsula was more potent than that collected from Egypt toward *E. coli* and *C. albicans* [9]. In the present study, it was found that mixing two different propolis potentiated the antimicrobial activity. The mechanism is not known. It might be related to synergistic effects of various compounds existed in the two different propolis samples.

It was found that the antimicrobial activity of propolis was due to bacterial cell membrane damage and cell lysis [26]. Furthermore, it has ability to inhibit bacterial motility, cell division, and protein synthesis by affecting RNA-polymerase [27]. Other studies demonstrated that propolis antimicrobial activities are mainly due to phenolic compounds, terpenes, caffeic, ferulic and coumaric acids, esters, and flavonoids [28,29].

The current study showed that the wound healing was faster in propolis A, propolis B, or mixed propolis-treated group than in the control group during the 25 days of the study. Interestingly, mixed propolis was more potent than propolis A or propolis B. Furthermore, the wound healing was faster with the use of propolis A compared to propolis B. This indicated that different samples of propolis have different wound healing properties.

The wound healing property of propolis is most likely due to well-known anti-inflammatory and antioxidants activities. It was found that propolis or its active ingredient, caffeic acid phenethyl ester (CAPE), inhibits the protein concentration of pro-inflammatory proteinase, cyclooxygenase (COX) activity, and prostaglandins, which results in stimulation of immune cells and phagocytes since prostaglandin is immunosuppressive [30-32]. Propolis inhibits eicosanoids and nitric oxide production, and it exhibits angiogenesis and anti-leukocyte activity, which explained its anti-inflammatory property [33]. Furthermore, propolis contains flavonoids and phenolic compounds, which have anti-inflammatory action [34]. They inhibited prostaglandin E2 production, COX-2, and mPGES-1 [35]. We have found that topical application of propolis normalizes the levels of IL-1β, IL-6, TNF-α, and MMP9 and significantly enhanced the production of collagen through the TGF-β1/Smad2,3 signaling axis in diabetic wound [2]. Recently, it was shown that propolis decreases neutrophils and macrophages in wound tissue, causes downregulation of the inflammatory transcription factor, nuclear factor kappa-light-chain-enhancer of activated B cells (pNF-κB) protein expression, and reduces production of TGF-β, TNF-α, and IL-6 [36]. Keratinocyte migration causes reepithelialization of the skin. It was found that propolis can accelerate the proliferation of skin keratinocytes [37].

Various parameters were used to assess the wound healing. Epithelialization and wound recovery are important parameters. Assessment of edema and redness, which are part of the cardinal sign of inflammation, also was conducted. All propolis samples reduced edema and redness, and the reduction was higher by mixed propolis. This effect is most likely due to anti-inflammatory property of propolis. Early reepithelialization results in faster wound closure that restores the integrity of the skin. This makes the wound less vulnerable to infection. The time required for complete epithelialization is important to assess the wound healing process and the efficacy of any intervention. Propolis A, propolis B, and mixed propolis enhanced epithelialization by increasing the amount of epithelial tissue covering the wounds, and they shorten the time for complete epithelialization. These observations were more obvious with the mixed propolis as compared to each propolis individually. This effect might be potentiated by mixing different propolis samples.

Wound healing is a complex process involving various cell types, cytokines, and intermingle stages. Hemostasis and inflammation are part of an inflammatory phase of wound healing, while granulation, contraction, and epithelialization are part of a proliferative phase of wound healing. Remodeling phase determines the wound appearance and strength.

The process of wound healing can be compromised by infection, inadequate oxygen supply, malnutrition, and oxidative process. In spite of major advances in our knowledge regarding the pathophysiology of the wound and wound healing, no treatment is available yet that can enhance or expedite wound healing process. Propolis can help wound healing most likely by its anti-inflammatory, antimicrobial, and antioxidative properties, which are essential for wound healing process. Damaged tissues provide an excellent media for microorganisms to grow and infect wound. In the current experiment, none of the wound treated by propolis showed any sign of infection.

Acceleration of the wound healing rate by mixed propolis might be due to the synergistic activity of active ingredients presented in the individual propolis samples when mixed. The antioxidant, anti-inflammatory, and antimicrobial activities of propolis make it a potential intervention in wound healing. Mixed propolis provides wound with more active ingredients, which result in a suitable environment for promoting healing process. Additive or synergistic activity provided by mixed propolis shortened the healing time and accelerated reepithelialization and wound recovery.

The results showed that not all propolis has the same potency to help wound healing and eradication of infection and mixing two different propolis samples resulted in a better antimicrobial and wound healing activity. Therefore, mixing different propolis samples collected from different regions, during different seasons, or from different bee species might produce propolis with higher biological activity. More studies are required to explore the potentiality of this approach, not limited to, in the management of microbial infection and wounds.

Mixing of different propolis samples might result in propolis with better antioxidant and anti-inflammatory activity. The two propolis samples were collected from different areas which are very much different in their predominant plants. Therefore, although the chemical composition of these two propolis samples was not done, basically their compositions should be different because of different plants predominant. However, it is important to analyze mixed propolis before and after being extracted. Furthermore, it is important to do the chemical analysis of the propolis before and after mixing, in particular, phenols, flavonoids, and CAPE ingredients. Studying the antioxidant and anti-inflammatory properties before and after mixing propolis samples is essential to explore whether mixing propolis can potentiate individual propolis anti-inflammatory and anti-oxidant capacity. Furthermore, a histological study of the wounds with and without propolis treatment will help to explain the mechanism of action. MIC, the lowest concentration of intervention that inhibits visible microbial growth, was used to evaluate the antimicrobial activity. It is regarded as a first step in the screening of activity against microorganisms in preclinical evaluation [38]. The macrobroth dilution assay was used where a dilution series of the propolis samples in broth was made in test tubes, and the microorganisms were added to each tube. The MIC can be performed using 96-well microdilution. However, using other methods is important such as minimum bactericidal concentration and Kirby–Bauer disk diffusion test. These investigations are currently in progress in our laboratory. Mixed propolis invention was submitted for a US patent in July 2017.

## Conclusion

The study showed for the first time that mixing different propolis samples collected from different geographical areas potentiates the wound healing properties and antimicrobial activity of propolis. A significant higher acceleration of wound reepithelialization and closure was obtained with the mixed propolis as compared with the individual propolis. This discovery is a breakthrough, and if it is confirmed by subsequent studies, it will have a favorable clinical and financial outcome.

## Authors’ Contributions

NA generated the idea, conducted the work in Iraq, wrote the paper, and submitted for publication.
